# Using Anthropometric Data and Physical Fitness Scores to Predict Selection in a National U19 Rugby Union Team †

**DOI:** 10.3390/ijerph18041499

**Published:** 2021-02-05

**Authors:** Luis Vaz, Wilbur Kraak, Marco Batista, Samuel Honório, Hélder Miguel Fernandes

**Affiliations:** 1 Department of Sport Science, Exercise and Health, School of Life and Environmental Sciences, University of Trás-os-Montes and Alto Douro, 5000-801 Vila Real, Portugal; 2 Research Centre in Sports Sciences, Health Sciences and Human Development (CIDESD), 5000-801 Vila Real, Portugal; hmfernandes@gmail.com; 3 Department of Sport Science, Faculty of Medicine and Health Sciences, Stellenbosch University, Stellenbosch 7600, South Africa; kjw@sun.ac.za; 4 Department of Sports and Well-Being, Polytechnic Institute of Castelo Branco, SHERU (Sport, Health and Exercise Research Unit), 6000-084 Castelo Branco, Portugal; marco.batista@ipcb.pt (M.B.); samuelhonorio@ipcb.pt (S.H.); 5 Research in Education and Community Intervention, RECI, 3500 Viseu, Portugal

**Keywords:** talent identification, rugby union, prediction of performance outcomes, selective factors.

## Abstract

The purpose of this study was to compare measures of anthropometry characteristics and physical fitness performance between rugby union players (17.9 ± 0.5 years old) recruited (n = 39) and non-recruited (n = 145) to the Portuguese under-19 (U19) national team, controlling for their playing position (forwards or backs). Standardized anthropometric, physical, and performance assessment tests included players’ body mass and height, push up and pull-up test, squat test, sit-and-reach test, 20 m shuttle run test, flexed arm hang test, Sargent test, handgrip strength test, Illinois agility test, and 20-m and 50-m sprint test. Results showed that recruited forwards players had better agility scores (*p* = 0.02, ES = −0.55) than the non-recruited forwards, whereas recruited backs players had higher right (*p* < 0.01, ES = 0.84) and left (*p* = 0.01, ES = 0.74) handgrip strength scores than their counterparts. Logistic regression showed that better agility (for the forwards) and right handgrip strength scores (for the backs) were the only variables significantly associated with an increased likelihood of being recruited to the national team. In sum, these findings suggest that certain well-developed physical qualities, namely, agility for the forwards players and upper-body strength for the back players, partially explain the selection of U19 rugby players to their national team.

## 1. Introduction

The expanded professionalism in rugby union has elicited fast changes in the athletic profile of rugby elite players [1]. Effective talent identification is an important issue in rugby union, as success at the international level conveys significant commercial benefits. Usually, the recruitment and development of rugby union players have focused on training interventions and game-play performance, with less attention on the anthropometric and physical performance characteristics [2]. However, the understanding of the rugby union movement patterns during competition, and the related physical demands, is critical to developing effective rugby training programs aiming to improve both athletes’ and teams’ performance [3]. Previous investigations have shown differences in fitness and morphological aspects between teams [4,5], levels of competition [6,7], and players’ positions [8,9].

According to this reasoning, some studies [10] reported that elite rugby players (backs and forwards) have specific skills, motor and physical abilities, and anthropometrical characteristics that distinguish them from the non-elite players. Under this scope, many studies have stressed the importance of anthropometric characteristics on team success in rugby union [11,12]. Indeed, previous studies demonstrated that teams with stronger players who possess heavier forwards and faster backs frequently achieve high success in elite international and club competitions; some physical fitness and player performance measures have also been found to be associated with game-related statistics [13]. Indeed, anthropometric characteristics, such as sprinting, tackling, muscular strength, and aerobic and anaerobic power, were reported as important factors to play at the elite level and illustrate the heterogeneous nature among rugby players.

The efficacy of training in youth and adolescent rugby players has been investigated [14], showing that substantial developments can be achieved with different approaches of effective rugby training programs to improve performance. However, the specific demands of competition differ markedly between forwards and backs [15], making it necessary to better understand these (potential) differences across the different playing positions.

Considering the important implications for rugby union, it is understandable that a degree of reluctance occurs concerning the explicit requirements in the prediction of performance outcomes in young talented rugby union players. Therefore, it is important to further investigate how rugby players’ anthropometry characteristics and physical and performance assessment tests can help to recruit, select, and develop rugby union elite players progression. The assessment of elite rugby players performance is frequently performed as part of their routine monitoring procedures, both to improve high-performance models for the elite player and monitor the player success according to training regimens. Considering the abovementioned information, the purpose of the current study is to compare measures of anthropometry characteristics and physical fitness performance between recruited and non-recruited rugby union players of under-19 (U19) national team, controlling for their playing position (forwards or backs).

## 2. Materials and Methods

### 2.1. Subjects

A total of 184 male junior rugby players from 2017/2018 Portuguese rugby academies were evaluated (age 17.9 ± 0.5 years old, height 1.79 ± 0.7 m, and body mass 84.2 ± 13.5 kg). Subjects were separated into two groups, based on recruited (n = 39) and non-recruited (n = 145), to represent the 2018 Portuguese rugby U19 national team, as well as according to their playing position (forwards or backs).

To be recruited, eligibility criteria to represent U19 national team included skills, selection policy, and principles established from “checklists” of technical and medical staff selectors (e.g., general knowledge and practical knowledge of the game, eligibility, time and availability, purpose of selection, personal and team profile ideas and systems, attributes, rugby experience, physical conditioning relative to rugby and specific positions, attitude, development, and game plan). All subjects were in the four-week pre-season period of their training plan, which included developing physical conditioning, strength and explosive power, acceleration, and power endurance and stamina. Throughout the preparation for the national competition, subjects performed 5 to 6 rugby training sessions with their clubs, usually 10 to 12 h weekly.

During the training camp week, all recruited players trained with the U19 national team twice a day (training volume of 4 to 6 h per day) and completed specific strength training sessions focused on the development of player´s physical conditioning.

After being fully informed about the experimental procedures, subjects agreed to participate in this study, and written guardian or parental consent was obtained before subjects were permitted to participate. Additionally, the subjects were informed that they were free to withdraw at any time without penalty.

None of the subjects reported any physical, psychological, or orthopedic limitation or injury that could limit their full participation. The study protocol was performed following the guidelines stated in the Declaration of Helsinki and was approved by the institutional University Ethical Committee (CE.UTAD 27/2016).

### 2.2. Anthropometric, Physical, and Performance Assessment Tests

Fitness testing results from the rugby elite development national academies representative squads were collected for each participant during the pre-season phase in the national camp each year. The total sample executed a set of anthropometric measures and physical tests on the initial day of the training camp week, as planned by the Portugal rugby U19 technical staff. All the tests were accomplished in a single day and under similar environmental conditions; the local temperature in October was 14.0 ± 2 °C, compared to 16.1 ± 2 °C in March. While the anthropometrics assessments started in the morning (≈9 a.m.) in a kinanthropometry laboratory, the physical performance assessment tests were held in the afternoon and were performed in outdoor natural turf rugby pitch and on running track (finished at ≈5 p.m.).

All assessments were carried out by medical and technical supervision of the staff of Portuguese rugby U19 team. Testing was led by competent technical staff who were adequately trained to use the devices and had the ability to perform the testing protocols.

A full dynamic warm-up drill with a gradual progression through a series of rugby movements was performed, including running at a moderate intensity, squats for 5-min, followed by 5-min of lunge twist active stretching and detailed exercises (e.g., submaximal jumps and accelerations before Sargent test, agility test, and acceleration and speed tests). After the warm-up (±3-min), players were required to perform the tests. Moreover, stretching exercises and a standardized recovery protocol were completed by all players after the final test sessions of the day. As part of these players’ training routines in rugby academies, all subjects were highly familiarized with the anthropometric and physical performance tests.

Players were allowed to ingest water during recovery periods (3 ± 2 min.). Anthropometrics measurements only included body height (m) and body mass (kg), whereas fitness testing battery included pull-up test, push up test, and free-standing squat [16]. The weight lifted and the number of repetitions done was used to calculate the 1RM [17]. The outdoor assessment tests included: Sargent test, flexed arm hang test, sit-and-reach test, and maximal aerobic power (20 m shuttle-run test) [18]. The VO_2_ max was estimated using regression equations described by [19]. Handgrip strength test (left and right hand) was performed as described by [20]. Acceleration and speed (10, 20, and 50 m sprint) using electronic timing gates and Illinois agility test (10 × 5 m) [21] were performed. A more detailed description of all tests may also be found in Table S1 (Supplementary Material). Test-retest reliability, typical error, and validity of measurement of all assessment tests used for this study have been previously confirmed in literature and their descriptions are documented elsewhere [22,23,24].

### 2.3. Procedures and Data Processing

Initially, data were inspected for normality using visual inspection (histograms and Q-Q plots of residuals) and analysis of skewness and kurtosis. Both procedures showed appropriate distributions and values.

Independent t-tests were used to identify significant differences between groups. Cohen’s *d* effect size statistics were calculated (ES), and respective 95% confidence intervals were also computed. Magnitudes of observed effects, and thresholds, were 0–0.20, trivial; 0.20–0.60, small; 0.60–1.20, moderate; 1.20–2.00, large; and >2.00, very large [25].

Multivariable forward logistic regression analyses [26] were performed separately for each playing position in order to identify potential significant predictors of being selected to the national team.

Statistical significance was set at *p* < 0.05, and calculations were carried out using SPSS software, 23.0, Version (IBM SPSS Statistics for Windows, Armonk, NY, USA: IBM Corp.).

## 3. Results

The descriptive results of the anthropometry characteristics and physical fitness measures are shown in Table 1 for the total sample and for playing positions (forwards and backs).

Forward players had greater body mass (*p* < 0.001, ES = 0.60), body height (*p* < 0.01, ES = 0.48), and push up performance (*p* = 0.04, ES = 0.31), whereas back players showed greater pull up (*p* < 0.01, ES = −0.49), left handgrip strength (*p* = 0.01, ES = −0.39), 20 m sprint (*p* < 0.001, ES = 1.26), 50 m sprint (*p* < 0.001, ES = 0.51), agility (*p* < 0.001, ES = 0.56), and flexibility performance (*p* < 0.001, ES = −0.53). No between-group significant differences were observed in the flexed arm hang (ES = −0.12), right handgrip strength (ES = −0.08), free standing squat (ES = 0.23), Sargent (ES = 0.05), and VO_2_max tests (ES = −0.15).

Table 2 presents the comparison results of the anthropometry characteristics and physical fitness scores between the recruited (n = 23) and non-recruited forwards (n = 83).

Descriptive and comparative results showed that the recruited forwards players had significantly better agility scores than their non-recruited counterparts, with a small to moderate size effect (*p* = 0.02, ES = −0.55). No other significant differences were observed between these groups.

Table 3 presents the comparison results of the anthropometry characteristics and physical fitness scores between the recruited (n = 16) and non-recruited backs (n = 62).

Results indicated that the recruited back players had significantly higher handgrip strength scores than their non-recruited counterparts. Both right (*p* < 0.01, ES = 0.84) and left (*p* = 0.01, ES = 0.74) values showed moderate size effects.

In order to better understand the potential effects of the anthropometry data and the physical fitness scores on the likelihood of rugby players being recruited for the U19 national team, separate logistic regressions were performed for each playing position.

With respect to the forward players, the logistic regression model was statistically significant (χ_(2)_ = 8.88, *p* = 0.01), explained 12.4% of the variance in the national team selection, and correctly classified 80.2% of cases. Better (lower) scores on the agility test were associated with an increased likelihood of being recruited to the national team (*β* = −0.56, *p* = 0.02).

When analyzing the back players, the logistic regression model was also statistically significant (χ_(1)_ = 7.48, *p* < 0.01), explained 14.3% of the variance in the national team selection, and correctly classified 82.1% of cases. Only better (higher) scores on the right handgrip strength test were associated with an increased likelihood of being recruited to the national team (*β* = 0.13, *p* < 0.01).

## 4. Discussion

This study aimed to quantify and explore how anthropometry and physical performance outcomes, according to playing position (forwards or backs), can help to recruit elite junior rugby union players. The results highlight an important association between handgrip strength and agility ability, and team recruitment in junior elite rugby union, and suggest that certain well-developed physical qualities, namely, agility for the forwards and upper-body strength for the backs, can help to predict the selection of U19 rugby players to their national team. Our findings are consistent with previous studies [27] describing forwards as having greater body mass and body height and exhibiting greater push-up performance when compared with faster, leaner, and smaller backs, who display greater values in 20 m and 50 m sprint, agility, and flexibility performance. No other significant differences were observed between these groups. The lack of clear differences illustrates the importance of supervision if the rugby development program aims to improve physical attributes. Therefore, physical characteristics should be developed from an early age to ensure the rugby player is physically ready for elite professional rugby union. Body composition, greater levels of strength and power, agility, and sprint ability appear to be important physical characteristics in rugby union players due to superior performances at higher playing levels and their relationship with game behaviors. Furthermore, the logistic regression results showed better agility scores for forwards and better right handgrip strength scores for backs. These variables were significantly associated with an increased likelihood of being recruited for the U19 Portugal team. The results indicate that hand strength can be considered an important capability and selective factor in elite junior rugby union players, especially considering that no previous study has examined the importance of the handgrip strength test in rugby players. In general, the evaluation of the importance of the handgrip strength used in rugby is not explored; however, hand dynamometry is simple, not expensive, and is a well-established method for assessing the strength of hand muscles.

In fact, the reliable evaluation of handgrip strength in rugby players appears to be an essential and somehow neglected component in strength monitoring, planning of strength training programs, as well as injury prevention and recovery. This test is suitable to assess players as it is a simple, inexpensive, and important diagnostic criterion. Frequently measured as a proxy for global muscle strength, this test is a well-established and recognized protocol and method for assessing hand strength [28].

In order to better understand the potential effects of the anthropometry data and the physical fitness scores on the likelihood of rugby players being recruited for the U19 national team, separate logistic regressions were performed for each playing position. Overall, the results were generally consistent with those of other rugby union studies [29,30] which demonstrated that different characteristics between forwards and backs players can be caused by specific player-position demands. Backs need to be leaner, faster, and aerobically fitter to defend in more open spaces, cover more distance during a rugby union match, perform more accelerations, and try to score more opportunities [31]. Conversely, forwards [32] need to be larger and stronger for scrumming with more force and to assist teammates winning line outs. Moreover, they need to gain and retain possession of the ball and receive more collisions (i.e., tackles, ball carries, and collisions).

Although we limited our investigation to the anthropometric characteristics and physical performances that discriminated between recruited and non-recruited rugby players according to playing position (forwards or backs) to U19 national team, it was previously reported that technical, tactical, and psychological skills were positively associated with game-specific position and performance measures; therefore, they can help to discriminate rugby elite players [33]. Indeed, the ability to make the appropriate decisions in game-play and during interactions between players is also a significant aspect of performance [34].

The non-use of motivation, learning effect, motor coordination, individual training status, and biomechanical factors (e.g., hand size) is another limitation of the current investigation. It is a possible claim that concentric or eccentric measures should have been evaluated rather than isometric strength. However, it should be noted that assessment of isometric strength can be easily obtained in practical contexts. To date, no studies have tested the possibility of using these study measures to understand this conflict’s effects to help improve the task of effective selection in elite junior rugby union players. Therefore, further research is required to understand the contribution of handgrip strength with other variables (e.g., physical training programs, player progress, strength control practices, and game behaviors). Lastly, a small sample size of subjects within each positional group (forwards and backs) would be useful for establishing a reliable testing protocol and/or the normative values for rugby players and to help selectors in the evaluation and recruitment of rugby union players.

## 5. Conclusions

In sum, the results of this study demonstrate the importance of handgrip strength and agility ability for the rugby national team selection process. Furthermore, objective measures can be useful for quantifying and evaluating player anthropometric characteristics and physical fitness performance progress. Thus, by combining further objective measures, rugby player recruitment and progress will be better monitored.

Indeed, the variables used allowed us to distinguish between recruited and non-recruited rugby union players for the Portugal U19 team. In addition to normal age-group criteria, subjective evaluation, or training and game perception aspects of performance, coaches can use this information to help in player recruitment approaches. The main findings have several implications for the effective selection process design, particularly by helping to identify and to improve the accuracy of elite junior rugby talent identification programs through a physical fitness assessment.

## Figures and Tables

**Table 1:** Descriptive statistics of the anthropometry characteristics and physical fitness performance measures for the total sample and for playing positions.

Variables	Total Sample (n = 184) M ± SD	Forwards (n = 106) M ± SD	Backs (n = 78) M ± SD
Body mass (kg)	84.28 ± 13.53	87.58 ± 13.38	79.81 ± 12.48
Body height (m)	1.79 ± 0.07	1.81 ± 0.07	1.78 ± 0.05
Push up test (reps)	43.25 ± 11.10	44.68 ± 12.00	41.30 ± 9.46
Pull up test (reps)	11.83 ± 4.29	10.96 ± 4.67	13.00 ± 3.40
Flexed arm hang test (s)	40.42 ± 13.63	39.75 ± 13.99	41.33 ± 13.15
Handgrip strength—right (kg)	48.00 ± 4.88	47.84 ± 4.16	48.21 ± 5.75
Handgrip strength—left (kg)	46.03 ± 4.65	45.28 ± 4.00	47.06 ± 5.27
Free standing squat (1RM)	140.77 ± 31.09	143.80 ± 31.89	136.67 ± 29.68
Sargent test (cm)	44.95 ± 5.06	45.07 ± 5.49	44.80 ± 4.45
20 m sprint (s)	2.99 ± 0.18	3.08 ± 0.13	2.89 ± 0.18
50 m sprint (s)	6.85 ± 0.27	6.91 ± 0.27	6.78 ± 0.26
Illinois agility test (s)	15.15 ± 0.96	15.37 ± 1.15	14.86 ± 0.49
Sit-and-reach test (cm)	32.93 ± 4.88	31.85 ± 5.26	34.38 ± 3.90
VO_2_max (mL kg^−1^ min^−1^)	49.69 ± 3.96	49.43 ± 3.93	50.03 ± 3.99

**Table 2:** Descriptive and comparative statistics of the anthropometry characteristics and physical fitness performance between recruited and non-recruited forwards.

Variables	Recruited (n = 23) M ± SD	Non-Recruited (n = 83) M ± SD	*p*	ES (95%CI)
Body mass (kg)	89.97 ± 14.36	86.91 ± 13.11	0.33	0.23 (−0.24, 0.69)
Body height (m)	1.82 ± 0.11	1.81 ± 0.07	0.31	0.24 (−0.22, 0.71)
Push up test (reps)	43.83 ± 14.56	44.92 ± 11.29	0.70	−0.09 (−0.55, 0.37)
Pull up test (reps)	11.78 ± 5.13	10.74 ± 4.54	0.34	0.22 (−0.24, 0.69)
Flexed arm hang test (s)	36.60 ± 10.44	40.62 ± 14.76	0.22	−0.29 (−0.75, 0.18)
Handgrip strength—right (kg)	48.53 ± 5.39	47.65 ± 3.76	0.38	0.21 (−0.25, 0.67)
Handgrip strength—left (kg)	46.35 ± 5.04	44.98 ± 3.64	0.15	0.34 (−0.12, 0.81)
Free standing squat (1RM)	140.57 ± 34.36	144.70 ± 31.33	0.59	−0.13 (−0.59, 0.33)
Sargent test (cm)	44.96 ± 4.71	45.10 ± 5.71	0.91	−0.03 (−0.49, 0.44)
20 m sprint (s)	3.05 ± 0.14	3.09 ± 0.13	0.16	−0.33 (−0.80, 0.13)
50 m sprint (s)	6.88 ± 0.31	6.92 ± 0.26	0.55	−0.14 (−0.60, 0.32)
Illinois agility test (s)	14.89 ± 1.40	15.50 ± 1.04	0.02	−0.55 (−1.01,−0.08)
Sit-and-reach test (cm)	32.09 ± 5.36	31.80 ± 5.26	0.81	0.06 (−0.41, 0.52)
VO_2_max (mL kg^−1^ min^−1^)	49.34 ± 4.77	49.46 ± 3.69	0.90	−0.03 (−0.49, 0.43)

**Table 3:** Descriptive and comparative statistics of the anthropometry characteristics and physical fitness performance between recruited and non-recruited backs.

Variables	Recruited (n = 16) M ± SD	Non-Recruited (n = 62) M ± SD	*p*	ES (95%CI)
Body mass (kg)	82.05 ± 8.38	79.23 ± 13.33	0.42	0.23 (−0.33, 0.78)
Body height (m)	1.77 ± 0.06	1.77 ± 0.05	0.69	−0.11 (−0.66, 0.44)
Push up test (reps)	40.94 ± 8.87	41.39 ± 9.67	0.87	−0.05 (−0.60, 0.50)
Pull up test (reps)	13.19 ± 3.64	12.95 ± 3.36	0.81	0.07 (−0.48, 0.62)
Flexed arm hang test (s)	42.00 ± 9.64	41.15 ± 13.97	0.82	0.06 (−0.49, 0.61)
Handgrip strength—right (kg)	51.85 ± 7.66	47.27 ± 4.78	<0.01	0.84 (0.27, 1.40)
Handgrip strength—left (kg)	50.03 ± 6.70	46.29 ± 4.59	0.01	0.74 (0.17, 1.30)
Free standing squat (1RM)	144.81 ± 26.95	134.57 ± 30.19	0.22	0.35 (−0.21, 0.90)
Sargent test (cm)	44.63 ± 6.00	44.84 ± 4.02	0.87	−0.05 (−0.60, 0.50)
20 m sprint (s)	2.86 ± 0.10	2.90 ± 0.19	0.47	−0.20 (−0.75, 0.35)
50 m sprint (s)	6.79 ± 0.15	6.77 ± 0.28	0.85	0.05 (−0.50, 0.60)
Illinois agility test (s)	14.78 ± 0.33	14.88 ± 0.53	0.49	−0.20 (−0.74, 0.36)
Sit-and-reach test (cm)	34.44 ± 3.25	34.37 ± 4.10	0.95	0.02 (−0.53, 0.57)
VO_2_max (mL kg^−1^ min^−1^)	50.01 ± 3.65	50.03 ± 4.11	0.99	−0.01 (−0.56, 0.54)

## Data Availability

The data presented in this study are available on request from the corresponding author. The data are not publicly available due to privacy and ethical restrictions.

## Supplementary Materials

The following are available online at https://www.mdpi.com/1660-4601/18/4/1499/s1, Table S1: Anthropometric, physiological and performance assessment test measurements/procedures.
