# Supplementary material for: Using Anthropometric Data and Physical Fitness Scores to Predict Selection in a National U19 Rugby Union Team
Source: Int J Environ Res Public Health. 2021 Feb 5;18(4):1499. doi: 10.3390/ijerph18041499 (PMC7915509; doi:10.3390/ijerph18041499)
Supplement: Supplementary file 1 [file ijerph-18-01499-s001.pdf]

Table 1. – Anthropometric, physiological and performance assessment test measurements/procedures. (adapted from Vaz, Vasilica, Carreras, Kraak, and Nakamura, 2016).

Test	Test measurements/Procedures
Anthropometric evaluation	Body weight (kg); Body height (m).
Push-up test	Procedure: A standard push-up begins with the hands and toes touching the floor, the body and legs in a straight line, feet slightly apart, the arms at shoulder width apart, extended and at a right angle to the body. Keeping the back and knees straight, the subject lowers the body to a predetermined point, to touch some other object, or until there is a 90-degree angle at the elbows, then returns back to the starting position with the arms extended. This action is repeated, and test continues until exhaustion, or until they can do no more in rhythm or have reached the target number of push-ups.
Pull-up test	The pull-up test (also called the chin-up test) is widely used as a measure of upper body strength and endurance. Procedure: The athletes grasped the overhead bar using either an overhand grip (palms facing away from body) or underhand grip (palms facing towards the body), with their arms fully extended. The subject then raised their body until their chin cleared the top of the bar and then lowered again to a position with their arms fully extended. The pull-ups had to be done in a smooth motion. Jerky motion, swinging the body, and kicking or bending the legs was not permitted. As many full pull-ups as possible were performed. The total number of correctly completed pull-ups was recorded. The type of grip was also recorded with the results.
Squat: free-standing squat (not on Smith machine)	The player lowered themselves in a controlled manner until the top of the thigh was parallel with the floor (knees flexed up to 80 degrees). A bungee cord or elastic band could be used across rack, which could be adjusted for each player to lower themselves to, to indicate appropriate depth. Players had to be proficient with the form before attempting heavy squats. Weight belts could not be used. For each test: The weight lifted and number of repetitions performed was used to calculate the players predicted 1RM using the following formula: *1RM = Weight lifted/(([Exp (-0.055 x Reps completed))] x 41.9 + 52.2/100*
Sargent test	The athlete stood side on to a wall and reached up with the hand closest to the wall. Keeping the feet flat on the ground, the point of the fingertips was marked or recorded. This is called the standing reach height. The athlete then stepped away from the wall, and leapt vertically as high as possible using both arms and legs to assist in projecting the body upwards. This jumping technique can use a countermovement. The athlete attempted to touch the wall at the highest point of the jump. The difference in distance between the standing reach height and the jump height was the score. The best of three attempts was recorded.
Flexed arm hang test	This test measures upper body relative strength and endurance. The participant climbed the ladder to a height so that their chin was level with the bar. They grasped the overhead bar using an overhand grip (palms facing away from body), with their hands shoulder width apart. On the command, "ready, go," the subject removed their feet from the ladder, and the timing started. The participant attempted to hold this position for as long as possible. Scoring: The total time in seconds was recorded—the timing was stopped when the subject's chin fell below the level of the bar or the head tilted backward to enable the chin to stay level with the bar.
Sit-and-reach test	The purpose of the test was to determine the joint range of motion and flexibility of the muscles around the hip joint using a sit and reach box with the “zero” point being 26 cm. The participant sat on the floor with knees extended (straight), and bare feet against the vertical edge of the sit-and-reach box. The participant then flexed (bent) at the hip and reached forward, with one hand placed over the top of the other, palms facing down, fingertips overlapping and elbows straight. Full stretch was held for at least 2 seconds to avoid the effect of bouncing. The furthest reach of the middle fingertips was recorded. The best of three attempts was recorded to the nearest 0.5 cm.

Table 1. – Anthropometric, physiological and performance assessment test measurements/procedures (adapted from Vaz, et al., 2016).

Test	Test measurements / Procedures
Maximal aerobic power (20m shuttle-run test)	Players were required to run back and forth (i.e., shuttle-run) along a 20m track, keeping in time with a series of signals on a compact disk. The frequency of the audible signals (and hence, running speed) was progressively increased, until subjects reached volitional exhaustion. Maximal aerobic power (VO_2max) was estimated using regression equations described by (Moore and Murphy, 2003)
Handgrip strength test (right and left hand).	Procedure: The subject held the dynamometer in the hand to be tested, with the arm at right angles and the elbow by the side of the body. The handle of the dynamometer was adjusted if required—the base rested on the first metacarpal (heel of palm), while the handle rested on the middle of the four fingers. When ready the subject squeezed the dynamometer with maximum isometric effort, which was maintained for about 5 seconds. No other body movement was allowed. The subject was strongly encouraged to give a maximum effort.
Acceleration and speed in a 20 and 50 m sprint effort	Players had a minimum of 20 minutes warm-up prior to testing which included a number of short maximal efforts. The running speed of players was evaluated with a 20 and 50 m sprint effort using dual beam electronic timing gates (swift performance equipment). The timing gates were positioned 20 and 50 m cross wind from a pre-determined starting point. Players were instructed to run as quickly as possible along the 50 m distance from a standing start. On a synthetic track, participants commenced the test in their own time, with timing starting once the beams of the first (0 m) timing gate were broken. Speed was measured to the nearest 0.01 s with the fastest value obtained from two trials used as the speed score.
Agility test: Illinois agility test	Procedure: The course was 10 meters long and 5 meters wide (distance between the start and finish points). Four cones were used to mark the start, finish and two turning points. Another four cones were placed down the center an equal distance apart. Each cone in the center was spaced 3.3 meters apart. Subjects lay on their front (head to the start line) and hands by their shoulders. On the 'Go' command the stopwatch was started, and the athlete got up as quickly as possible and ran around the course in the direction indicated, without knocking the cones over, to the finish line, at which point the timing was stopped.

Moore, A., & Murphy, A. (2003). Development of an anaerobic capacity test for field sport athletes. *J Sci Med Sport, 6*(3), pp. 275-284. Retrieved from https://www.ncbi.nlm.nih.gov/pubmed/14609144

Vaz, L., Vasilica, I., Carreras, D., Kraak, W., & Nakamura, F. Y. (2016). Physical fitness profiles of elite under-19 rugby union players. *J Sports Med Phys Fitness, 56*(4), pp. 415-421. Retrieved from https://www.ncbi.nlm.nih.gov/pubmed/25651896
